# Temporary Bridge Plating vs Primary Arthrodesis of the First Tarsometatarsal Joint in Lisfranc Injuries: Randomized Controlled Trial

**DOI:** 10.1177/1071100720925815

**Published:** 2020-06-05

**Authors:** Are H. Stødle, Kjetil H. Hvaal, Helga M. Brøgger, Jan Erik Madsen, Elisabeth Ellingsen Husebye

**Affiliations:** 1 Division of Orthopaedic Surgery, Oslo University Hospital, Oslo, Norway; 2 Department of Radiology and Nuclear medicine, Oslo University Hospital, Oslo, Norway; 3 Institute of Clinical Medicine, University of Oslo, Oslo, Norway

**Keywords:** Lisfranc injury, tarsometatarsal joint injury, primary arthrodesis, bridge plate

## Abstract

**Background:**

Unstable Lisfranc injuries are best treated with anatomic reduction and stable fixation. There are controversies regarding which type of stabilization is best. In the present study, we compared primary arthrodesis of the first tarsometatarsal (TMT) joint to temporary bridge plating in unstable Lisfranc injuries.

**Methods:**

Forty-eight patients with Lisfranc injuries were included and followed for 2 years. Twenty-four patients were randomized to primary arthrodesis (PA) of the medial 3 TMT joints, whereas 24 patients were randomized to temporary bridge plate (BP) over the first TMT joint and primary arthrodesis of the second and third TMT joints. The main outcome parameter was the American Orthopaedic Foot & Ankle Society (AOFAS) midfoot scale and the secondary outcome parameters were the 36-Item Short Form Health Survey (SF-36) and visual analog scale for pain (VAS pain). Computed tomography (CT) scans pre- and postoperatively were obtained. Radiographs were obtained at follow-ups. Pedobarographic examination was performed at the 2-year follow-up. Twenty-two of 24 patients in the PA and 23/24 in the BP group completed the 2-year follow-up.

**Results:**

The mean AOFAS midfoot score 2 years postoperatively was 89 (SD 9) in the PA group and 85 (SD 15) in the BP group (*P* = .32). There were no significant differences between the groups with regard to SF-36 or VAS pain scores. The alignment of the first metatarsal was better in the BP group than in the PA group measured by the anteroposterior Meary angle (*P* = .04). The PA group had a reduced peak pressure under the fifth metatarsal (*P* = .047). In the BP group, 11/24 patients had radiologic signs of osteoarthritis in the first TMT joint.

**Conclusion:**

Both treatment groups had good outcome scores. The first metatarsal was better aligned in the BP group; however, there was a high incidence of radiographic osteoarthritis in this group.

**Level of Evidence:**

Therapeutic level I, prospective randomized controlled study.

## Introduction

Lisfranc injuries are defined as injuries to the tarsometatarsal joint complex, which includes the tarsometatarsal (TMT) joints, intercuneiform and naviculocuneiform joints.^16,21^ These injuries consist of a wide spectrum of injuries from nondisplaced, stable injuries to severe fracture dislocations.^6,21,24,31,32,35^ Several studies have shown anatomic reduction and stable fixation to be the most important factors in achieving a good functional outcome in acute Lisfranc fracture dislocations.^2,5,14,17,23,28,36^ The best technique used to achieve an anatomic and stable fixation of the joints is still debated. Open reduction and transarticular screw fixation has been the standard approach for many years.^2,3,14,21,32,36^ In recent years, good results have been reported on dorsal bridge plating of the TMT joints after Lisfranc injuries.^1,9,11,17,33^ In contrast to transarticular screw fixation, the dorsal bridge plate technique avoids additional damage to the cartilage and might reduce the high incidence of post-traumatic osteoarthritis in these patients.^1,9,11,14,20,25,32^ Primary arthrodesis of the 3 medial TMT joints has also been advocated in treating unstable Lisfranc injuries. Two randomized controlled trials comparing transarticular screw fixation to primary arthrodesis in high-energy Lisfranc injuries have shown favorable results in the primary arthrodesis group.^8,18^ In addition, one retrospective study comparing primary arthrodesis to transarticular screw fixation in young patients with low-energy Lisfranc injuries also showed favorable results in the arthrodesis group.^4^

In the present study, we have compared the first TMT joint–preserving technique using a temporary dorsal bridge plate, to primary arthrodesis in acute Lisfranc injuries. A primary arthrodesis of the second and third TMT joint was performed in all patients.

Our hypothesis was that the joint-sparing procedure using a temporary dorsal bridge plate would lead to a better functional outcome compared to primary arthrodesis.

## Methods

**Figure 1.**
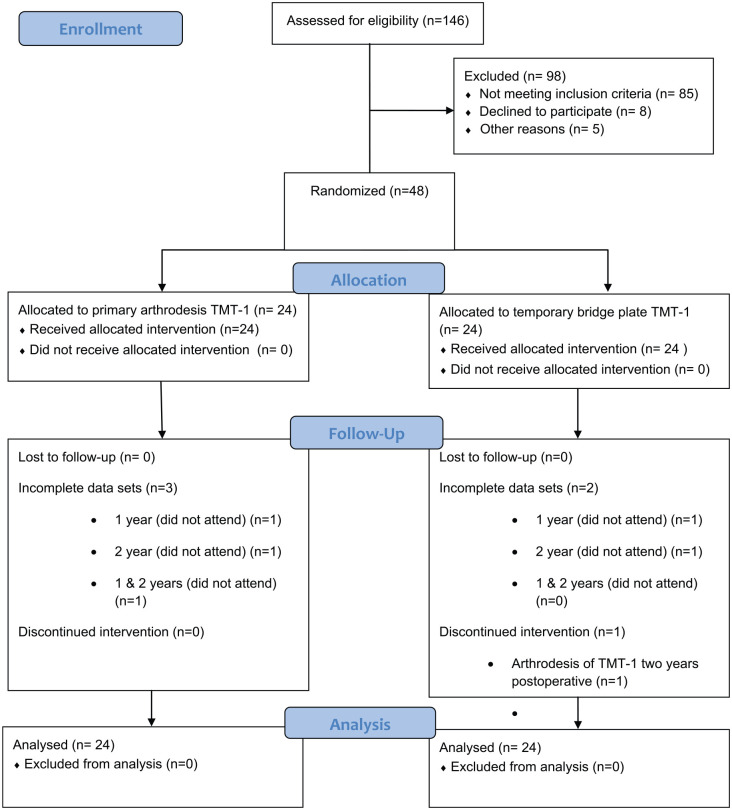
CONSORT flowchart of the trial enrollment and analysis. TMT, tarsometatarsal.

A randomized controlled trial comparing primary arthrodesis (PA) to temporary bridge plating (BP) of the first TMT joint in acute Lisfranc injuries was conducted (Figure 1). The study was approved by the National Committee for Medical and Health Research, registered in Clinical trials.org (ID: NCT01448941), and approved by the data protection officer at the university hospital. All patients signed an informed consent form prior to inclusion. The patients were enrolled from October 2011 to August 2015 (Figure 1). The trial was performed at a level 1 trauma center.

An acute Lisfranc injury was defined as injury to the TMT joints with avulsion fractures, intra-articular fractures, and/or displacement of 1 or more TMT joints. Injuries to the TMT joint were identified using primary radiographs, CT scans, stress fluoroscopy, and/or weightbearing radiographs.

The joints were considered unstable if radiographs or CT scans showed a displacement of 2 mm or more in any direction or a stress test under fluoroscopy showed displacement of the joints.

Inclusion criteria were Lisfranc injuries with instability of the medial 3 TMT joints and no fractures in relation to the first TMT joint, in patients between 18 and 65 years old. Minor capsular avulsions in relation to the first TMT joint was accepted as a primarily ligamentous injury.

The exclusion criteria were concomitant other major lower extremity injuries / polytrauma, open injuries, previous foot pathology, diabetes mellitus, neuropathy, and peripheral vascular disease.

### Randomization

After informed consent was obtained, the patients were randomly assigned to one of the 2 groups using the random allocation rule. Allocations were kept in sealed, opaque envelopes containing “Primary Arthrodesis” or “Bridge Plate” and were manually shuffled. Neither the orthopedic surgeon performing the follow-up examinations nor the patients were blinded.

#### Demographics

Twenty-four patients were randomized to the BP group, and 24 patients were randomized to the PA group. The patient characteristics at time of inclusion, mechanisms of injury, and injury characteristics are presented in Table 1. No between-group differences were observed at baseline, except from a higher rate of ligamentous injuries in the lateral column in the BP group (*P* = .049). The time from injury to surgery was 16.5 days in the PA group and 15 days in the BP group (*P* = .68). The bridge plate over the first TMT joint in the BP group was removed at a mean of 138 (SD 22) days after primary surgery. The “homerun-screw” and the hardware used for the TMT joint arthrodesis were not routinely removed.

**Table 1.** Patient Characteristics at Time of Enrollment, Mechanism of Injury, and Injury Characteristics.

Characteristics	PA (n= 24)	BP (n=24)
Gender, male/female	11/13	11/13
Age^a^	30 (23-40)	34 (28-40)
Side, right/left	13/11	7/17
Smoking, yes/no	3/21	5/19
BMI^a^	24 (21-28)	25 (22-27)
Mechanism of injury		
Fall <1 m or twisting of foot	12	7
Fall 1-3 m	2	1
Fall >3 m	0	1
Crush injury	0	1
Bike accident	0	2
Sports-related injury	10	12
Injury characteristics		
Ligamentous injuries		
Medial column	24	24
Central column	2	6
Lateral column^b^	2	9
Intra-articular fractures		
Medial column	0	0
Central column	22	18
Lateral column^b^	15	9

Abbreviations: BP, bridge plate; PA, primary arthrodesis.

a Presented as median and interquartile range in parentheses.

b The only statistically significant difference between the PA and BP group at enrollment was a higher rate of ligamentous injuries in the lateral column in the BP group, *P* = .049.

### Operative Technique and Rehabilitation

**Figure 2.**
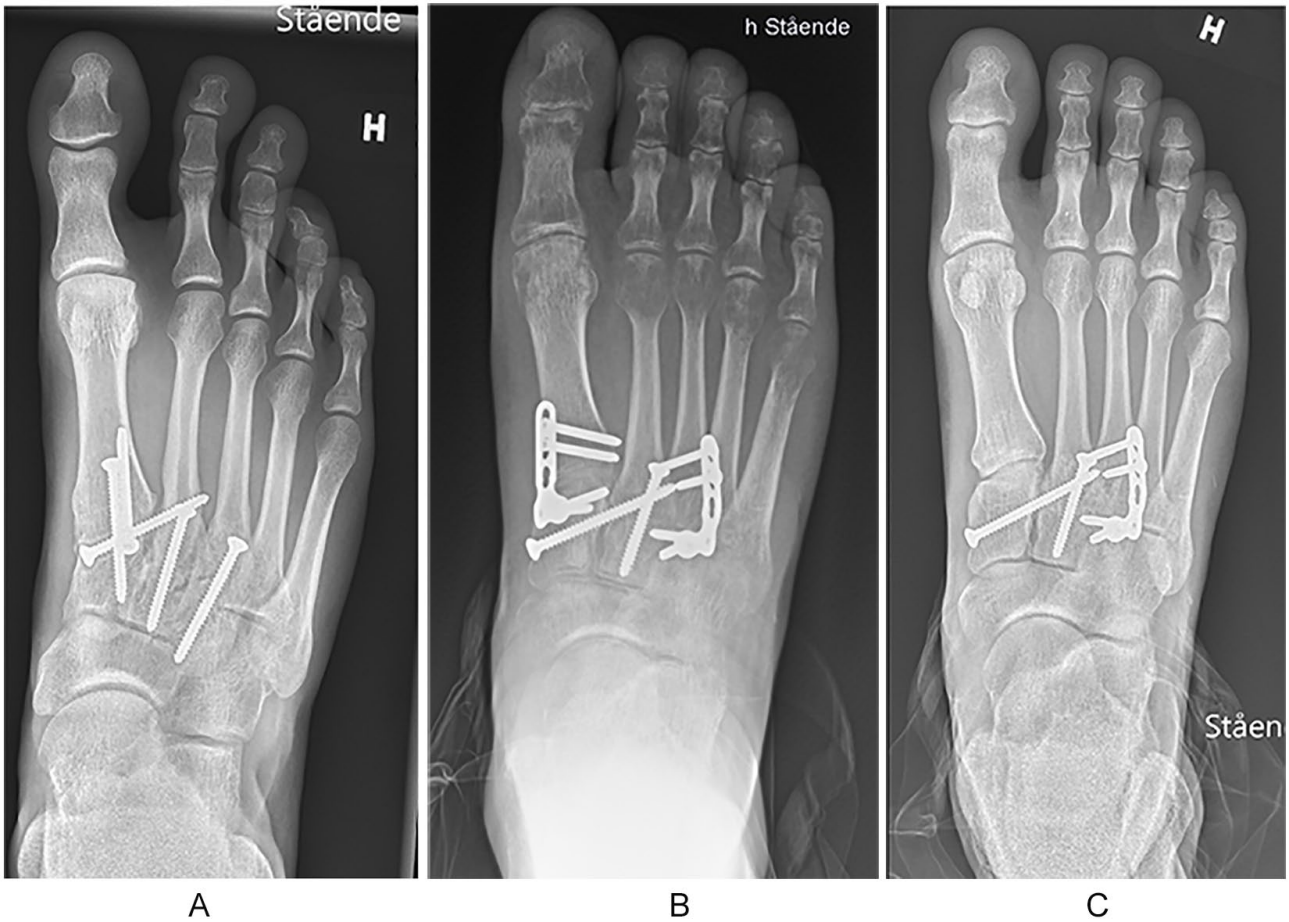
(A) Primary arthrodesis of the 3 medial TMT joints. (B) Temporary bridge plating of the first TMT joint using locking plate and primary arthrodesis of the second and third TMT joint. (C) Same patient as in panel B, but after removal of the temporary bridging plate. TMT, tarsometatarsal.

The surgeries were performed by orthopedic surgeons experienced in foot and ankle surgery. A 2-incision technique was used, with one longitudinal dorsomedial incision over the first TMT joint and a second incision over the third TMT joint. A skin bridge of at least 4 cm between the incisions was preserved to reduce the risk of wound complications. The 3 medial TMT joints were exposed. As all of the patients were treated with a primary arthrodesis of the second and third TMT joint, the cartilage was removed from these joints and the subchondral bone was multiperforated using a 2-mm drill bit to enhance fusion. No bone graft was used. If the patient was randomized to the PA group, the same procedure was performed in the first TMT joint. In the patients randomized to dorsal bridge plating, the cartilage of the first TMT joint was left intact. The TMT joints were then reduced anatomically, starting with the first TMT joint and temporary fixed using 1.6-mm Kirschner wires. The reduction was confirmed both visually and by fluoroscopy. In the BP group, the first TMT joint was bridged with a 2.7-mm locking plate (LCP Compact Foot 2.4/2.7; DePuy Synthes, Oberdorf, Switzerland), while in the arthrodesis group the arthrodesis of the first TMT joint was fixed with two 2.7- or 3.5-mm fully threaded screws with interfragmentary compression. The primary arthrodesis of the second and third TMT joints was either fixed using 2.7- or 3.5-mm fully threaded screws or, in case of a severely comminuted joint, a locking plate was used. A “homerun-screw” was then placed from the medial cuneiform to the base of the second metatarsal securing the Lisfranc mortise. After reduction and fixation of the 3 medial TMT joints, the reduction of the fourth and fifth TMT joints was assessed. If displaced, the 2 lateral TMT joints were reduced and stabilized using 1.6-mm Kirschner wires (Figure 2).

### Postoperative Care

The patients remained non–weight bearing for the first 6-8 weeks. Postoperatively the patients had a short leg splint that was removed at 2-3 weeks together with the sutures, and a short leg cast was applied. Cast immobilization was discontinued at 6-8 weeks postoperatively, and any K-wires transfixing the fourth and fifth TMT joints were removed. The patients then started weight-bearing as tolerated in a walker boot, which was used during weight-bearing until 12 weeks after surgery. In the BP group, the dorsal bridge plate over the first TMT joint was removed 4-5 months after surgery.

### Outcome Measures

The patients returned for follow-up at 2-3 weeks, 6-8 weeks, 12 weeks, 6 months, 1 and 2 years postoperatively. The main outcome measure was the American Orthopaedic Foot & Ankle Society (AOFAS) midfoot scale, which consists of 3 main parts (pain, function, and alignment) resulting in a score ranging from 0 to 100, the best score being 100.^12^ The secondary outcome parameters where the visual analog scale (VAS) for pain at rest and during walking, and the 36-Item Short Form Health Survey (SF-36) health survey, a patient-reported survey of patient health, at the 1- and 2-year follow-ups.^35^ The same orthopedic surgeon conducted all of the 1- and 2-year follow-up examinations.

### Radiographic Measurements

Postoperative CT scans and radiographs were obtained within the first 3 days after surgery. Weight-bearing anteroposterior (AP), lateral, and 30-degree oblique radiographs were obtained at the 6-week, 12-week, 12-month, and 24-month follow-ups. Reduction was evaluated on the postoperative CT scans. An anatomic reduction was defined as <2-mm displacement in any direction of the TMT joints, and a distance of no more than 2 mm between the medial cuneiform and the base of the second metatarsal.

At the 2-year follow-up, the alignment of the foot was assessed with weight-bearing radiographs. A displacement of 2 mm or more compared with the postoperative CT scans was considered as secondary displacement. The Meary angle (talus-first metatarsal angle) was measured on the AP and lateral weightbearing radiographs to assess any difference in the alignment of the first metatarsal between the 2 groups.^15^ Osteoarthritis (reduced joint space, sclerosis, cysts, or osteophytes) of the first TMT joint in the BP group, as well as any nonunion of the fused TMT joints, were recorded.

### Pedobarography

At 2-year follow-up, the plantar pressure of the patients was evaluated with the Tekscan HR mat (Tekscan Inc, South Boston, MA) and the Tekscan research software. Contact length, peak pressure and contact area were recorded for the following areas: medial heel (MH), lateral heel (LH), midfoot (MF), first metatarsal (M1), second metatarsal (M2), third metatarsal (M3), fourth metatarsal (M4), fifth metatarsal (M5), and first toe (T1).

### Statistical Analysis

A superiority study was conducted and power analysis prior to the study enrollment showed that 44 patients (22 in each group) had to be included to show a difference of 10 points or more on the AOFAS scale, with an estimated standard deviation of 10. This would give a power of 0.9 and a significance level of 5%. Taking into account any loss to follow-up we planned for 24 patients included in each group. The statistical analyses were performed using the Statistical Package for the Social Science (SPSS) software, version 25 (SPSS Inc, Chicago, IL). Data were tested for normality. Normal distributed data are presented as means with standard deviations and the 2-sided *t* test for independent samples was used for statistical analysis. The Mann-Whitney *U* test was used for nonparametric data, which are presented as median and interquartile range (IQR). The primary outcome (AOFAS midfoot scale), which was used for power analysis, showed a nonparametric distribution. These data are presented as means and standard deviation (SD), and the *t* test has been applied. In addition, the data were analyzed using nonparametric tests (Mann-Whitney *U* test), gaining similar results. The Fischer exact test was used to analyze categorical data. Spearman rank correlation was used to calculate correlation between variables. The significance level was *P* ≤ .05.

## Results

### Clinical Outcomes

The mean AOFAS midfoot score and median VAS pain scores were not significantly different between the 2 groups at the 1- and 2-year follow-up (Table 2).

**Table 2.** Outcome Measures.

Outcome Measure	PA	BP	*P* Value
AOFAS midfoot scale^a^			
1 y (PA: n=23, BP: n=23)	85 (10)	79 (16)	.12
2 y (PA: n=22, BP: n=23)	89 (9)	85 (15.0)	.32
VAS pain at rest^b^			
1 y (PA: n=23, BP: n=23)	0 (0-0)	0 (0-2.4)	.14
2 y (PA: n=22, BP: n=23)	0 (0-0.4)	0 (0-0.8)	.72
VAS pain during walking^b^			
1 y (PA: n=23, BP: n=23)	1.4 (0-4.2)	2.5 (0-4.4)	.45
2 y (PA: n=22, BP: n =23)	0 (0-2.0)	0.9 (0-3.0)	.42
SF-36 1-y^b^ (PA: n=23, BP: n=23)			
Physical function	90 (85-100)	90 (69-96)	.44
Role physical	100 (75-100)	88 (25-100)	.05
Bodily pain	90 (70-90)	70 (45-90)	.18
General health	80 (65-95)	80 (65-90)	.91
Vitality	60 (45-75)	60 (54-66)	.62
Social function	100 (63-100)	100 (72-100)	.93
Role emotional	100 (67-100)	100 (0-100)	.29
Mental health	80 (48-80)	80 (50-80)	.62
Physical component	54 (39-54)	51 (33-51)	.27
Mental component	54 (30-54)	54 (31-54)	.46
SF-36 2-y^b^ (PA: n=22, BP: n=22)			
Physical function	95 (89-100)	95 (89-100)	.98
Role physical	100 (44-100)	100 (88-100)	.61
Bodily pain	90 (62-100)	90 (82-100)	>.99
General health	83 (70-95)	75 (64-90)	.25
Vitality	60 (44-75)	63 (45-71)	.88
Social function	94 (72-100)	100 (84-100)	.37
Role emotional	100 (25-100)	100 (58-100)	.54
Mental health	78 (60-93)	80 (72-84)	.69
Physical component	55 (49-58)	54 (46-58)	.92
Mental component	50 (36-58)	52 (47-55)	.75

Abbreviations: AOFAS, American Orthopaedic Foot & Ankle Society midfoot scale; BP, bridge plate; IQR, interquartile range; PA, primary arthrodesis; SF-36, 36-Item Short Form Health Survey; VAS pain, visual analog scale for pain.

a Data are presented as mean, with the SD in parentheses.

b Data are presented as median, with the IQR in parentheses.

The SF-36 at one- and 2-year follow-up did not show any significant difference between the groups in any of the 8 subgroups nor in the physical and mental component summary (Table 2).

### Radiographic Outcomes

The radiographic findings are presented in Table 3. In both the PA and the BP group, 21 of the 24 patients had an anatomical reduction evaluated on the postoperative CT scan. At the 2-year follow-up, no patients had secondary displacement on weightbearing radiographs. In the PA group, the AP Meary angle was significantly increased compared with the BP group (12 degrees; IQR, 5-15 vs 5 degrees; IQR, 2-13, *P* = .04).

**Table 3.** Radiographic Findings.

Radiographic Findings	PA Group	BP Group	*P* Value
K-wire fixation TMT 4+5	6 (24)	2 (24)	.245
Anatomic reduction postop CT scan^a^	21 (24)	21 (24)	>.99
Anatomic reduction radiographs at 2 y	21 (22)	23 (23)^b^	.49
AP Meary angle, degrees, median (IQR)	12 (5-15)	5 (2-13)	**.042** ^d^
Lateral Meary angle, degrees, median (IQR)	1 (0-3)	3 (0-6)	.23
Nonunion (all third TMT joint)	1 (22)	2 (23)	>.99
Osteoarthritis TMT-1		11 (24)^c^	
Spontaneous fusion TMT-1		4 (23)	

Abbreviations: AP, anteroposterior; BP, bridge plate; CT, computed tomography; IQR, interquartile range; PA, primary arthrodesis; TMT, tarsometatarsal.

a There was 1 nonanatomic reduction in the first TMT joint in each group.

b Two of the TMT joint displacements seen on the postoperative CT scans were not detected on the 2-year follow-up radiographs.

c The one patient who did not attend the 2-year follow-up had osteoarthritis of the first TMT joint on the 1-year follow-up.

d Bold indicates statistically significant findings.

After 2 years, 11/24 patients in the BP group had radiographic osteoarthritis in the first TMT joint. Only 1 of these patients required conversion to arthrodesis of the first TMT joint at 24 months because of painful osteoarthritis.

### Pedobarography

**Figure 3.**
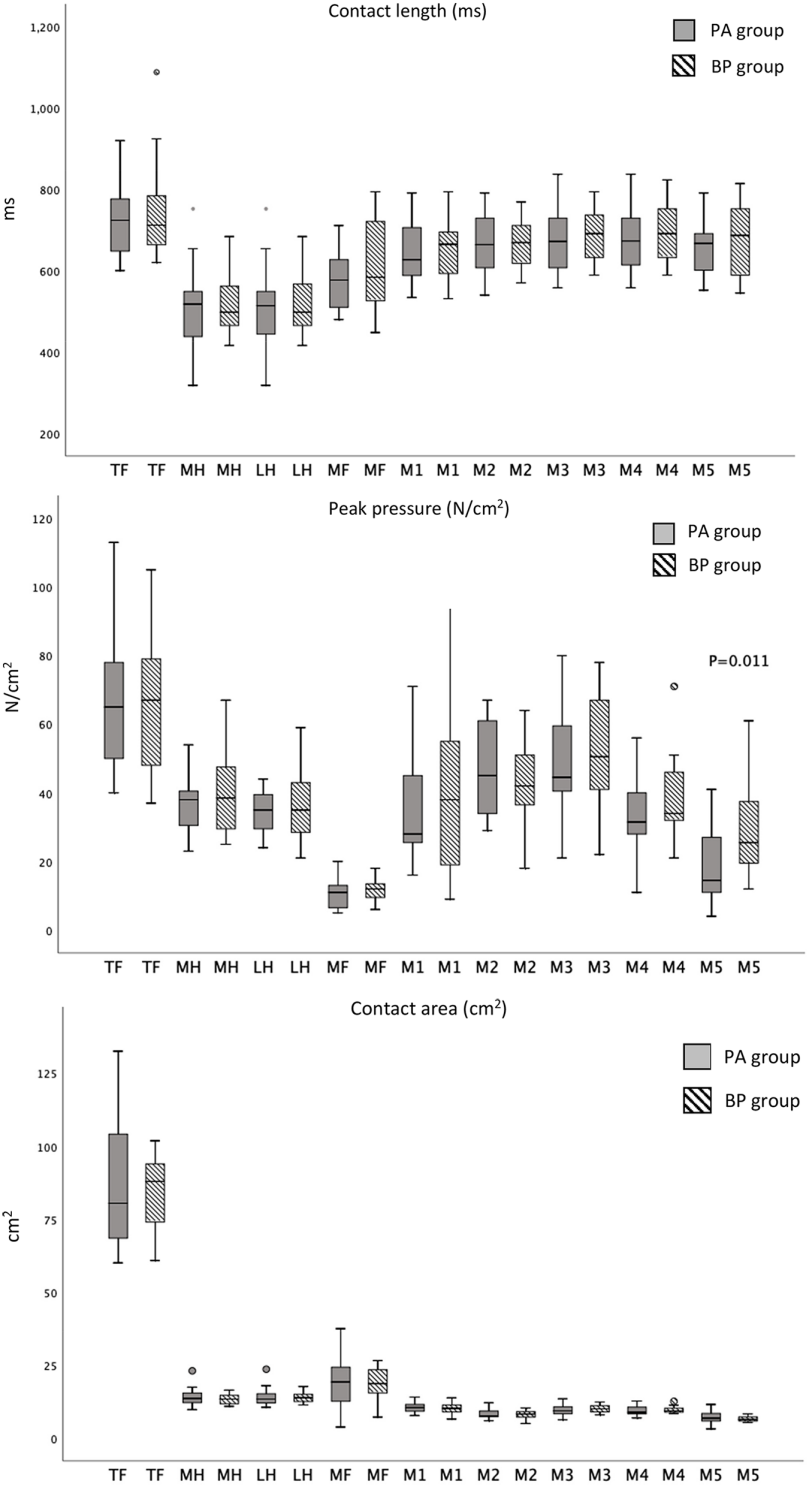
Pedobarographic data of injured side presented as boxplots. The only statistically significant finding (Mann-Whitney *U* test) was the reduced peak pressure under the fifth metatarsal in the PA group (*P* = .01). BP, bridge plate; M1, first metatarsal; M2, second metatarsal; M3, third metatarsal; M4, fourth metatarsal; M5, fifth metatarsal; MF, midfoot; MH, medial heel; LH, lateral heel; PA, primary arthrodesis; TF, total foot.

The pedobarographic results are presented in Figure 3. The only statistically significant difference between the 2 groups was the reduced peak pressure under the fifth metatarsal in the PA group. The peak pressure under the fifth metatarsal was also reduced compared with the noninjured side in the PA group (16 N/cm^2^, IQR, 13-24, vs 26 N/cm^2^, IQR, 15-31; *P* = .047); this reduction was not seen in the BP group. The fifth metatarsal peak pressure was correlated to the lateral Meary angle in the PA group (*r*_s_ = −0.65, *P* = .002).

### Complications and Additional Surgeries

One patient had a superficial wound infection in the BP group treated successfully with oral antibiotics. Two patients in the BP group developed complex regional pain syndrome. In the PA group, 1 patient had persistent pain because of irritation of the medial branch of the superficial peroneal nerve. One patient in the BP group experienced loosening of a screw from the plate, which led to a rupture of the extensor hallucis longus tendon. The patient was treated with a transfer of the second toe extensor digitorum longus to extensor hallucis longus with an excellent outcome. No statistical difference in complication rate was observed between the 2 groups (*P* = .348).

Nonunions developed in the third TMT joint in 3 patients (1 in the PA and 2 in the BP group). In addition to the planned removal of the bridge plate in the BP group, 7/23 patients in the PA and 5/24 patients in the BP group had hardware removed (*P* = .450) at a median of 302 days (IQR, 149-725).

## Discussion

In this randomized controlled trial comparing temporary bridge plating (BP) to primary arthrodesis (PA) of the first TMT joint in acute Lisfranc injuries, we were not able to detect superiority of the BP group compared to the PA group using AOFAS midfoot score, VAS pain score, or SF-36 at 2-year follow-up. A high incidence of radiologic osteoarthritis was present in the first TMT joint in the BP group; however, only 1 patient required a fusion due to painful osteoarthritis. The first metatarsal was better aligned in the AP radiographs in the BP group evaluated by the AP Meary angle. Pedobarographic examination revealed a reduced peak pressure under the fifth metatarsal in the PA group. We had a trend toward more complications in the BP group (*P* = .348).

The medial 3 TMT joints are considered nonessential joints, and fusion of these joints is well tolerated by patients.^8,13,18,19,22^ The first tarsometatarsal joint is more mobile than the central TMT joints, and preserving this joint might be beneficial.^21^ As there is almost no motion in the second and third TMT joints and these joints have a tendency to develop painful osteoarthritis, these joints were fused in the present study.^13^ The lateral column is the most mobile and plays an important role in the foot’s ability to adapt to the surface and should not be fused.^13,20,22^ In the current study, we have compared preserving the first TMT joint to fusing it.

We did not find superiority of the BP group compared to the PA group with regards to the AOFAS midfoot score or VAS pain score (mean AOFAS 89 vs 85, *P* = .32 and median VAS pain during walking 0 vs 1.0, *P* = .42). Both groups had good outcome scores, comparable to the study by Ly et al, in which the primary arthrodesis group had a mean AOFAS midfoot score of 88 and VAS pain score of 1.2 at 2-year follow-up.^18^ No studies have, to our knowledge, investigated the outcome after combining temporary bridge plating of the first TMT joint with primary arthrodesis of the second and third TMT joints. However, the AOFAS midfoot scores were similar to the results of temporary bridge plating reported by Hu et al in their prospective comparative study with an average follow-up of 31 months (mean AOFAS midfoot score in plate group of 83.1), and in the retrospective study by Kirzner et al (mean AOFAS midfoot score in plate group of 82.5).^9,11^ Stern and Assal reported a mean AOFAS midfoot score of 85 at 1-year follow-up in their retrospective case series of 15 patients treated with dorsal bridge plating.^33^ In all these studies the first, second, and/or third TMT joints were stabilized using bridge plates whereas the 2 lateral TMT joints where stabilized using K-wires, if unstable.

With regard to quality of life, Schepers et al reported normalization of the SF-36 score at a median follow-up of 76 months after isolated Lisfranc injuries.^30^ The SF-36 score in both groups of our study was comparable to reported data from the Norwegian general population aged 30-39 years, both with regard to the physical component summary (mean 53, SD 8) and mental component summery (mean 52, SD 9) score, suggesting a return to quality of life at the same level as the general population.^7^

Osteoarthritis after Lisfranc injuries is reported in up to 25%-94%.^14,20,25,32^ The bridge plate technique is a joint-sparing technique that might reduce the risk of osteoarthritis.^1,11,33^ Lau et al was, however, not able to relate osteoarthritis to the method of fixation when comparing bridge plating to transarticular screw fixation^17^ Previous studies using dorsal bridge plating have reported a high rate of mild post-traumatic osteoarthritis on follow-up radiographs.^17,33^ In the present study 11/24 patients had degenerative changes on the follow-up radiographs at 2 years, but only 1 patient required an arthrodesis of the first TMT joint due to pain. The long-term incidence and severity of osteoarthritis after temporary bridge plating, and how many of these patients will require an arthrodesis, are still not known.

Hu et al reported a spontaneous fusion rate of 5/32 (15,6%) after treating Lisfranc injuries with joint-preserving dorsal bridge plates, but 4 of the patients did not have any discomfort.^9^ In the present study 4/24 patients treated with a bridge plate of the first TMT joint developed a spontaneous fusion of the joint within 2 years. The patients with a spontaneous fusion had a favorable outcome with a median AOFAS midfoot score at 2 years of 92 (range, 80-100).

In the 2 previously published randomized controlled trials comparing primary arthrodesis to transarticular screw fixation in Lisfranc injuries, low rates of nonunions were reported (1/21 and 1/19).^8,18^ Altogether, there were 3 nonunions after arthrodesis in our present study, all of these occurred in the third TMT joint (one in the PA group and 2 in the BP group). These patients had only minor complaints and did not want further surgery with revision arthrodesis.

Anatomic reduction of the TMT joints and a stable fixation are considered the most important factors in achieving a good outcome when treating unstable Lisfranc injuries.^11,14,23,29,32^ Kirzner et al found a trend toward better reduction of Lisfranc injuries using bridge plating compared with transarticular screw fixation (*P* = .06).^11^ On the other hand, Lau et al were not able to confirm any differences in the quality of reduction when comparing transarticular screw fixation to bridge plating.^17^ In the present study, we could not find a difference between the 2 groups in the anatomic reduction of the TMT joints or in the distance between the medial cuneiform and the base of the second metatarsal on postoperative CT scan or on the radiographs at the 2-year follow-up. However, examining the alignment of the first metatarsal we did observe an increased AP Meary angle in the PA group compared to the BP group (12 vs 5 degrees, *P* = .042), indicating that the first metatarsal was fixed in an abducted position. Lamm et al reported the AP Meary angle in normal subjects to have a median value of 6.5 degrees (range, 0-18) which is comparable to the findings in our BP group.^15^ No statistical difference was found between the 2 groups when comparing the lateral Meary angle, although the PA group had a slightly reduced angle compared with the BP group (1 vs 3 degrees, *P* = .23). Pedobarographic examination showed a reduced fifth metatarsal peak pressure in the PA group compared with the BP group (*P* = .047). The fifth metatarsal peak pressure correlated to the lateral Meary angle in the PA group (*r*_s_ = −0.65, *P* = .002). In addition, the medial column is shortened when performing an arthrodesis, which also may elevate the head of the first metatarsal. Both the shortening of the medial column and the slightly reduced lateral Meary angle can lead to increased pronation of the foot. We believe that the reduced fifth metatarsal pressure was caused by a change in the length and alignment of the first metatarsal in the PA group.

Loss of reduction has been reported as a complication to bridge plating of Lisfranc injuries. Kirzner and colleagues reported a loss of reduction in 11% of their patients treated with bridge plates vs 24% in the group treated with transarticular screws.^11^ We found no patients with a secondary displacement at the 2-year follow-up. This might be because only the first TMT joint was bridge plated and the second and third TMT joints were fused, in contrast to the bridge plate group in the study by Kirzner, where all the unstable central and medial joints were bridge plated.^11^

All the patients in the BP group had the locking plate bridging the first TMT joint removed at a mean of 138 (22) days after the primary surgery. This additional surgery can be one of the causes for the higher number of complications, although not statistically significant, seen in the BP group.

The strength of the present study is first and foremost its randomized controlled design. The study is, to our knowledge, the only study comparing temporary bridge plating to primary arthrodesis in Lisfranc injuries. All the surgeries were performed by orthopedic surgeons experienced in foot and ankle surgery and the surgeons were familiar with both operative techniques prior to study inclusion. Anatomic reduction was evaluated with postoperative CT scans, and there was a low rate of loss to follow-up in the study.

The study does have some inherent limitations. A majority of the patients sustained low-energy trauma as polytrauma patients were excluded. Low-energy injuries are known to have better results than the high-energy injuries in terms of outcome scores, postoperative reduction, and post-traumatic osteoarthritis.^17,27,34^ Only the patients with clinically and/or radiologically suspected nonunion were evaluated with CT scans. We thereby might have missed nonunions with only minor symptoms. The patients and examiner were not blinded for the treatment, with a potential for introducing bias. In addition, the AOFAS midfoot scale has been shown to have an acceptable validity and internal consistency when used to evaluate Lisfranc injuries, but has a ceiling effect and a lack of coverage and targeting.^26^ Even so, a review of patient-reported outcome measures in foot and ankle surgery showed the AOFAS midfoot scale to be the most commonly used outcome measure in studies of Lisfranc injuries together with the VAS for pain and SF-36.^10^ This makes these 3 outcome scores suitable for comparing the results with previously published studies.

## Conclusion

Both the temporary bridge plate group and the primary arthrodesis group yielded good outcome scores when treating Lisfranc injuries. We did not find superiority of the BP group compared to the PA group according to the AOFAS midfoot score. The first metatarsal was better aligned in the BP group. Despite avoiding transarticular screw damage by bridge plating the first TMT joints, there was a high rate of radiologically detected osteoarthritis in the first TMT joint. The long-term effects of post-traumatic osteoarthritis is still unknown and longer follow-up is required.

## Supplemental Material

FAI925815_disclosures – Supplemental material for Temporary Bridge Plating vs Primary Arthrodesis of the First Tarsometatarsal Joint in Lisfranc Injuries: Randomized Controlled Trial Click here for additional data file. Supplemental material, FAI925815_disclosures for Temporary Bridge Plating vs Primary Arthrodesis of the First Tarsometatarsal Joint in Lisfranc Injuries: Randomized Controlled Trial by Are H. Stødle, Kjetil H. Hvaal, Helga M. Br�gger, Jan Erik Madsen and Elisabeth Ellingsen Husebye in Foot & Ankle International

## References

[1] Alberta FG Aronow MS Barrero M Diaz-Doran V Sullivan RJ Adams DJ. Ligamentous Lisfranc joint injuries: a biomechanical comparison of dorsal plate and transarticular screw fixation. Foot Ankle Int. 2005;26(6):462-473. 15960913 10.1177/107110070502600607

[2] Arntz CT Veith RG Hansen ST, et al Fractures and fracture-dislocations of the tarsometatarsal joint. J Bone Joint Surg Am. 1988;70(2):173-181. 3273882

[3] Benirschke SK Kramer PA. High energy acute lisfranc fractures and dislocations. Tech Foot Ankle Surg. 2010;9(3):82-91.

[4] Cochran G Renninger C Tompane T Bellamy J Kuhn K. Primary arthrodesis versus open reduction and internal fixation for low-energy lisfranc injuries in a young athletic population. Foot Ankle Int. 2017;38(9):957-963. 28602113 10.1177/1071100717711483

[5] Coetzee JC. Making sense of lisfranc injuries. Foot Ankle Clin. 2008;13(4):695-704. 19013403 10.1016/j.fcl.2008.07.001

[6] Curtis MJ Myerson M Szura B. Tarsometatarsal joint injuries in the athlete. Am J Sports Med. 1993;21(4):497-502. 8368407 10.1177/036354659302100403

[7] Garratt AM Stavem K. Measurement properties and normative data for the Norwegian SF-36: results from a general population survey. Health Qual Life Outcomes. 2017;15(1):1-10. 28292292 10.1186/s12955-017-0625-9 PMC5351285

[8] Henning JA Jones CB Sietsema DL Bohay DR Anderson JG. Open reduction internal fixation versus primary arthrodesis for lisfranc injuries: a prospective randomized study. Foot Ankle Int. 2009;30(10):913-922. 19796583 10.3113/FAI.2009.0913

[9] Hu S Chang S Li X Yu G. Outcome comparison of Lisfranc injuries treated through dorsal plate fixation versus screw fixation. Acta Ortop Bras. 2014;22(6):315-320. 25538478 10.1590/1413-78522014220600576 PMC4273957

[10] Hunt KJ Hurwit D. Use of patient-reported outcome measures in foot and ankle research. J Bone Joint Surg Am. 2013;95(16):e118(1-9). 10.2106/JBJS.L.01476 23965711

[11] Kirzner N Zotov P Goldbloom D Curry H Bedi H. Dorsal bridge plating or transarticular screws for Lisfranc fracture dislocations. Bone Joint J. 2018;100-B(4):468-474. 29629578 10.1302/0301-620X.100B4.BJJ-2017-0899.R2 PMC6503757

[12] Kitaoka HB Alexander IJ Adelaar RS Nunley JA Myerson MS Sanders M. Clinical rating systems for the ankle-hindfoot, midfoot, hallux, and lesser toes. Foot Ankle Int. 1994;15(7):349-353. 7951968 10.1177/107110079401500701

[13] Komenda GA Myerson MS Biddinger KR. Results of arthrodesis of the tarsometatarsal joints after traumatic injury. J Bone Joint Surg Am. 1996;78:1665-1676. 8934480 10.2106/00004623-199611000-00005

[14] Kuo RS Tejwani NC Digiovanni CW, et al Outcome after open reduction and internal fixation of Lisfranc joint injuries. J Bone Joint Surg Am. 2000;82-A:1609-1618. 10.2106/00004623-200011000-00015 11097452

[15] Lamm BM Stasko PA Gesheff MG Bhave A. Normal foot and ankle radiographic angles, measurements, and reference points. J Foot Ankle Surg. 2016;55(5):991-998. 27320694 10.1053/j.jfas.2016.05.005

[16] Lau S Bozin M Thillainadesan T. Lisfranc fracture dislocation: a review of a commonly missed injury of the midfoot. Emerg Med J. 2017;34(1):52-56. 27013521 10.1136/emermed-2015-205317

[17] Lau S Howells N Millar M De Villiers D Joseph S Oppy A. Plates, screws, or combination? Radiologic outcomes after lisfranc fracture dislocation. J Foot Ankle Surg. 2016;55(4):799-802. 27079306 10.1053/j.jfas.2016.03.002

[18] Ly TV Coetzee JC. Treatment of primarily ligamentous lisfranc joint injuries: primary arthrodesis compared with open reduction and internal fixation: A prospective, randomized study. J Bone Joint Surg. 2006;88(3):514. 16510816 10.2106/JBJS.E.00228

[19] Mulier T Haan J Vriesendorp P Reynders P. The treatment of lisfranc injuries: review of current literature. Eur J Trauma Emerg Surg. 2010;36(3):206-216. 26815863 10.1007/s00068-010-1034-5

[20] Mulier T Reynders P Dereymaeker G Broos P. Severe Lisfrancs injuries: primary arthrodesis or ORIF? Foot ankle Int. 2002;23(10):902-905. 12398141 10.1177/107110070202301003

[21] Myerson MS. The diagnosis and treatment of injury to the tarsometatarsal joint complex. J Bone Joint Surg Br. 1999;81(5):756-763. 10530832 10.1302/0301-620x.81b5.10369

[22] Myerson MS Cerrato RA. Current management of tarsometatarsal injuries in the athlete. J Bone Joint Surg Am. 2008;90(11):2522-2533. 18978422

[23] Myerson MS Fisher RT Burgess AR Kenzora JE. Fracture dislocations of the tarsometatarsal joints: end results correlated with pathology and treatment. Foot Ankle. 1986;6(5):225-242. 3710321 10.1177/107110078600600504

[24] Nunley JA Vertullo CJ. Classification, investigation, and management of midfoot sprains. Am J Sports Med. 2002;30(6):871-878. 12435655 10.1177/03635465020300061901

[25] Pereira CJ Espinosa EG Miranda I Pereira MB Canto RSDT Evaluation of the surgical treatment of lisfranc joint fracture-dislocation. Acta Orthop Bras. 2008;16:93-97.

[26] Ponkilainen VT Uimonen M Repo JP Mattila VM Haapasalo HH. Validity and internal consistency of the American Orthopaedic Foot & Ankle Society Midfoot Scale in patients with Lisfranc injury [published online ahead of print June 21, 2019]. Foot Ankle Surg. 2019. 10.1016/j.fas.2019.06.005 31255577

[27] Renninger CH Cochran G Tompane T Bellamy J Kuhn K. Injury characteristics of low-energy lisfranc injuries compared with high-energy injuries. Foot Ankle Int. 2017;38(9):964-969. 28693353 10.1177/1071100717709575

[28] Richter M Thermann H Huefner T Schmidt U Krettek C. Aetiology, treatment and outcome in Lisfranc joint dislocations and fracture dislocations. Foot Ankle Surg. 2002;8(1):21-32.

[29] Richter M Wippermann B Krettek C Schratt HE Hufner T Thermann H. Fractures and fracture dislocations of the midfoot: Occurrence, causes and long-term results. Foot Ankle Int. 2001;22(5):392-398. 11428757 10.1177/107110070102200506

[30] Schepers T Kieboom BCT Kieboom B van Diggele P Patka P Van Lieshout EMM Pedobarographic analysis and quality of life after Lisfranc fracture dislocation. Foot Ankle Int. 2010;31(10):857-864. 20964963 10.3113/FAI.2010.0857

[31] Scolaro J Ahn J Mehta S. Lisfranc fracture dislocations. Clin Orthop Relat Res. 2011;469(7):2078-2080. 20878282 10.1007/s11999-010-1586-z PMC3111796

[32] Stavlas P Roberts CS Xypnitos FN Giannoudis PV. The role of reduction and internal fixation of Lisfranc fracture-dislocations: a systematic review of the literature. Int Orthop. 2010;34(8):1083-1091. 20683593 10.1007/s00264-010-1101-x PMC2989076

[33] Stern RE Assal M. Dorsal multiple plating without routine transarticular screws for fixation of lisfranc injury. Orthopedics. 2014;37(12):815-819. 25437072 10.3928/01477447-20141124-03

[34] van Koperen PJ de Jong VM Luitse JSK Schepers T. Functional outcomes after temporary bridging with locking plates in lisfranc injuries. J Foot Ankle Surg. 2016;55(5):922-926. 27267412 10.1053/j.jfas.2016.04.005

[35] Ware JE Sherbourne CD The MOS 36-item short-form health survey (SF-36). I. Conceptual framework and item selection. Med Care. 1992;30(6):473-483. 1593914

[36] Watson TS Shurnas PS Denker J. Treatment of Lisfranc joint injury: current concepts. J Am Acad Orthop Surg. 2010;18(12):718-728. 21119138 10.5435/00124635-201012000-00002

